# STA-Mediated Interferometry with a Single Trapped Particle

**DOI:** 10.3390/e28030267

**Published:** 2026-02-28

**Authors:** Alvaro Rodriguez-Prieto, Sofía Martínez-Garaot, Ion Lizuain

**Affiliations:** 1 Department of Applied Mathematics, University of the Basque Country EHU, 48040 Leioa, Spain; ion.lizuain@ehu.eus; 2 EHU Quantum Center, University of the Basque Country EHU, 48040 Leioa, Spain; sofia.martinez@ehu.eus; 3 Department of Physical Chemistry, University of the Basque Country EHU, 48040 Leioa, Spain

**Keywords:** shortcuts to adiabaticity (STA), interferometry, trapped particles, inverse engineering

## Abstract

We reviewand update schemes for different measurements using STA-mediated guided interferometry with a single trapped particle. STA stands for “shortcuts to adiabaticity”, a set of techniques to achieve the results of adiabatic dynamics in shorter times. In the first scheme we presented a protocol aimed at detecting weak unknown forces. It consisted of a single ion trapped in a harmonic potential and driven by time-and-spin-dependent forces generated via off-resonant lasers. Our approach provided stability and the independence of the results on the motional states for the small-oscillations regime. We could, also, design faster-than-adiabatic processes with sensitivity control. However, it required a rotation of the trapping potential at the moment the experiment starts. A much more practical and broadly applicable design was then developed, where no rotation is involved. Here, a single atom is driven by two moving spin-dependent trapping potentials where we guide the arms of the interferometer via shortcuts to adiabatic paths. In this paper, in addition to a brief review of these two previous proposals, we revisit the first scheme and present a new protocol where the spin-dependent driving force is generated via a “shaken” optical lattice. This opens the possibility for additional interferometric measurements beyond an unknown force, for example, the mass of the trapped ion, while still preserving the advantages of the previously proposed method.

## 1. Introduction

Atom interferometry [1,2] works by first splitting and later recombining the atomic wavefunction, allowing us to detect the differential phase accumulated during the separation. This phase differential is very sensitive to weak potential differences between the arms of the interferometer. It has been shown to obtain impressive sensitivities for high-precision measurements in velocity and inertial sensors, gravimeters or gyrometers. Several systems are currently being investigated which involve cold atoms in optical lattices [3,4] and where wave function branches are separated by internal-state-dependent potentials [5,6,7].

Particularly, in a previous work [8] we designed an ion-driven interferometer to measure weak unknown forces. We applied inverse engineering techniques with the help of Lewis–Riesenfeld invariants of motion [9]. Here, a fixed harmonic trap was combined with two homogeneous time- and spin-dependent driving forces, which are generated via an optical lattice and designed to separate first and recombine at final time the wave function branches of the trapped ion. These driving forces are applied at $x_{0}\left(t\right)$, which plays the role as the “crossing point” of the potential energies for the spin-dependent forces. The resulting phase differential at final time, which is proportional to an unknown force *c*, depends on $x_{0}$. Within this scheme, we got stability properties such as the independence of the final phase with respect to motional excitations and the geometric character of the phase. It is also noteworthy that the sensitivity of the interferometer may be chosen at will, subjected to technical limitations.

However, if the unknown force *c* acts permanently, even before the experiment starts at t=0, the minimum of the harmonic trap, $V\left(x,t\right)=\frac{1}{2}m\omega^{2}x^{2}$, is shifted from 0 to $c/m\omega^{2}$, which becomes the “natural” choice for $x_{0}$. As a result, no differential phase arises and *c* can not be measured. A formal but hardly practical solution is to rotate the effectively one-dimensional trap from a perpendicular position exactly when the experiment starts, so we let the force *c* act only from t=0 onward.

To avoid such difficulty, we worked out a different setting [10] again using STA-mediated guided interferometry [6,8,10,11]. Operationally, this proposal differs from the previous one. Here, we use two moving spin-dependent traps, not necessarily harmonic, complemented by homogeneous spin- and time-dependent forces to compensate for inertial terms due to the motion of the traps [12]. This compensation can be equivalently found by invariant-based inverse engineering, by the “fast-forward approach” [13] or as a local unitary transformation of a nonlocal counterdiabatic approach [14]. Another quantum control protocol which accelerates the adiabatic transport of trapped ions has been recently proposed [15].

The phase differential within this new setting is independent of the $x_{0}$ pivot equipotential point. Therefore, no rotation of the trap is needed, so this scheme is more broadly applicable. In addition, using arbitrary potentials rather than harmonic ones opens the way to using ultracold neutral atoms where the anharmonicities are usually stronger than for trapped ions.

In both previous schemes our aim was measuring a weak unknown force. In this paper, in addition to a brief review of these proposed two protocols to detect weak forces, we present a new scheme whose aim is rather to measure the mass of an ion trapped in a harmonic potential. Therefore, this work opens new possibilities for STA-mediated interferometry.

The new scheme is very similar to the first one [8] but differs from it because we consider that the driving spin-dependent force is generated via a “shaken” optical lattice [16,17], which induces an oscillating $x_{0}\left(t\right)$. Despite this difference, it still preserves advantages such as the independence of the results from the the motional states for the small oscillation regime.

We study in detail the phases accumulated along branches and derive an expression for the phase differential at final time which is proportional to the mass of the trapped ion. We also explore how to inverse engineer the driving force so we get the desired trajectories, allowing a better measurement of the mass of the trapped ion.

The paper is organized as follows. In Section 2 we introduce the basic principles of the interferometer. In Section 3 we present two different schemes aimed at measuring unknown weak forces. For both we first analyze the phases accumulated along the branches and, later, make use of the Lewis–Riesenfeld invariants of motions to inverse engineer the trap trajectories. In Section 4 we consider a shaking optical lattice generating an oscillating $x_{0}\left(t\right)$. We analyze the phases along the branches and study, in detail, how to inverse engineer appropriate trajectories and driving forces.

## 2. The Interferometer

Our setting involves a single particle with two internal states: “spin up”, |↑〉, and “spin down”, |↓〉. The particle state can be written as

(1) $$|\Psi \left(t\right)\rangle =a_{↑}\left|↑\rangle \right|\psi_{↑}\left(t\right)\rangle +a_{↓}\left|↓\rangle \right|\psi_{↓}\left(t\right)\rangle ,$$

where $|\psi_{↑}\left(t\right)\rangle$ and $|\psi_{↓}\left(t\right)\rangle$ are the motional states for the two internal levels. We assume a prepared state from which a π/2 pulse [18] produces two equally weighted components ($a_{↑}=a_{↓}=1/\sqrt{2}$). At t=0, immediately after the π/2 pulse, and assuming a Lamb–Dicke regime, $|\psi_{↑}\left(0\right)\rangle =|\psi_{↓}\left(0\right)\rangle$. The two branches are then driven by designed spin-dependent forces and evolve separately. At the final time $t_{f}$, we have

(2) $$\langle \psi_{↓}\left(t_{f}\right)|\psi_{↑}\left(t_{f}\right)\rangle =e^{i\Delta \phi (t_{f})}\left|\langle \psi_{↓}\left(t_{f}\right)|\psi_{↑}\left(t_{f}\right)\rangle \right|.$$

A second π/2 pulse gives the populations

(3) $$P_{↑↓}\left(t_{f}\right)=\frac{1}{2}\pm \frac{1}{2}ℜe\left[\langle \psi_{↓}\left(t_{f}\right)|\psi_{↑}\left(t_{f}\right)\rangle \right]=\frac{1}{2}\pm \frac{1}{2}\cos\Delta \phi \left(t_{f}\right),$$

where we inverse-engineer the driving forces such that $|\langle \psi_{↑}\left(t_{f}\right)|\psi_{↓}\left(t_{f}\right)\rangle |=1$. An unknown force *c* can be measured from the populations if the differential phase $\Delta \phi (t_{f})$ is proportional to *c*, $\Delta \phi (t_{f})=S\cdot c$, where the sensitivity *S* is known. In such a case, *c* may be found unambiguously from the periodicity 2π/c of the $P_{↑↓}\left(t_{f}\right)$ as a function of *S* [8]. As the interferometer works with a single particle, measuring the populations requires repetitions in time. Similarly, the mass of the trapped particle, *m*, might be measured if $\Delta \phi (t_{f})$ is proportional to *m* instead of *c*, $\Delta \phi \left(t_{f}\right)=S^{*}\cdot m$, where the sensitivity $S^{*}$ is known.

## 3. Measuring a Weak Unknown Force

In the first configuration [8], we consider a single ion of mass *m* and charge *e* trapped in a radially tight, effectively one-dimensional harmonic trap. A weak constant unknown force *c* that we want to measure acts on the ion in the longitudinal direction. In addition, the ion is subjected to “spin-dependent” driving forces opposite for the two internal levels, $f\left(t,\sigma_{z}\right)=\sigma^{z}f\left(t\right)=\pm f\left(t\right)$. These forces are generated by off-resonant lasers, and the Lamb–Dicke regime is assumed [19] so that these forces might be considered homogeneous.

The Hamiltonians for the two arms of the interferometer read

(4) $$\begin{array}{ccc} H^{↑↓} & = & \frac{p^{2}}{2m}+\frac{1}{2}m\omega^{2}x^{2}-cx\mp f\left(t\right)\left[x-x_{0}\left(t\right)\right]=\\ & = & \frac{p^{2}}{2m}+\frac{1}{2}m\omega^{2}{\tilde{x}}^{2}\mp f\left(t\right)\tilde{x}-\frac{c}{2m\omega^{2}}\left[c\pm 2f(t)\right]\pm f\left(t\right)x_{0}\left(t\right), \end{array}$$

where we have introduced $\tilde{x}=x-\frac{c}{m\omega^{2}}$. Solving the Schrödinger equations for each of the Hamiltonians, we get the wavefunctions

(5) $$\begin{array}{ccc} \psi_{↑↓}^{c\neq 0}\left(\tilde{x},t\right) & = & e^{\frac{i}{\hbar }\frac{c}{2m\omega^{2}}\int_{0}^{t}\left[c\pm 2f\left(t^{\prime }\right)dt^{\prime }\right]}\\ & & \times e^{\mp \frac{i}{\hbar }\int_{0}^{t}x_{0}\left(t^{\prime }\right)f\left(t^{\prime }\right)dt^{\prime }}\psi_{↑↓}^{c=x_{0}=0}\left(\tilde{x},t\right), \end{array}$$

where $\psi_{↑↓}^{c=x_{0}=0}\left(\tilde{x},t\right)$ are the solutions for the system whose Hamiltonians are $H^{↑↓}$ with c=0 and $x_{0}=0$. The phases accumulated when traveling through each of the branches are

(6) $$\phi_{↑↓}\left(t\right)=\frac{c}{2\hbar m\omega^{2}}\int_{0}^{t}\left[c\pm 2f_{\alpha }\left(t^{\prime }\right)\right]dt^{\prime }\mp \frac{1}{\hbar }\int_{0}^{t}x_{0}\left(t^{\prime }\right)f_{\alpha }\left(t^{\prime }\right)dt^{\prime }$$

where $f_{\alpha }\left(t\right)$ is designed inversely from the Newton equation

(7) $$\ddot{\alpha }\left(t\right)+\omega^{2}\alpha \left(t\right)=\frac{f_{\alpha }\left(t\right)}{m},$$

for particular solutions α(t) that satisfy the boundary conditions $\alpha \left(t_{b}\right)=\dot{\alpha }\left(t_{b}\right)=\ddot{\alpha }\left(t_{b}\right)=0$ for $t_{b}=0,t_{f}$. Therefore, $\int_{0}^{t_{f}}f_{\alpha }\left(t\right)dt=m\omega^{2}\int_{0}^{t_{f}}\alpha \left(t\right)dt$ and the phase differential at final time is

(8) $$\Delta \phi \left(t_{f}\right)=\phi_{↑}-\phi_{↓}=\frac{2c}{\hbar }\int_{0}^{t_{f}}\alpha \left(t\right)dt-\frac{2}{\hbar }\int_{0}^{t_{f}}x_{0}\left(t\right)f_{\alpha }\left(t\right)dt.$$

For a constant $x_{0}$,

(9) $$\Delta \phi \left(t_{f}\right)=\frac{2}{\hbar }\left[c-m\omega^{2}x_{0}\right]\int_{0}^{t_{f}}\alpha \left(t\right)dt.$$

If *c* acts on the ion at t≤0, even before the driving force is applied, the minimum of the trapping potential is shifted from 0 to $c/m\omega^{2}$. Thus, the spin-dependent potential terms will be naturally centered so that they cross at that point. This means that $x_{0}=c/m\omega^{2}$ becomes the most “natural” choice for applying the driving forces, which leads to $\Delta \phi (t_{f})=0$. However, if we rotate the trap from a perpendicular direction at t=0, *c* starts to act on the ion exactly at that moment, so the minimum of the trap is still at $x_{0}=0$ and

(10) $$\Delta \phi \left(t_{f}\right)=\frac{2c}{\hbar }\int_{0}^{t_{f}}\alpha \left(t\right)dt=S\cdot c.$$

Here $S=\frac{2}{\hbar }\int_{0}^{t_{f}}\alpha \left(t\right)dt$ is the sensitivity of the interferometer. Please note that we may increase the sensitivity at will (subjected to technical limitations) by increasing the area under the trajectory followed by the trapped ion. Rotating the trap then becomes a formal but hardly practical solution.

### 3.1. Inverse-Engineering Techniques

Here we make use of the Lewis–Riesenfeld invariants of motion to inverse engineer the trap trajectories. Our aim is ensuring that the final states meet again at a chosen final time at their original positions and without any residual excitations. In our particular case we have a forced harmonic oscillator. For the spin-up branch, it reads

(11) $$H=\frac{p^{2}}{2m}+\frac{1}{2}m\omega^{2}x^{2}-f_{\alpha }\left(t\right)x,$$

for which we can find a quadratic invariant

(12) $$I=\frac{1}{2m}{\left(p-m\dot{\alpha }\right)}^{2}+\frac{1}{2}m\omega^{2}{\left(x-\alpha \right)}^{2}$$

where α(t) satisfies the Newton Equation (7) with boundary conditions, as explained above. Solving

(13) $$I\left(t\right)\psi_{n}\left(x,t\right)=\lambda_{n}\psi_{n}\left(x,t\right)$$

we get both the eigenvalues and the eigenvectors:

(14) $$\begin{array}{ccc} & \lambda_{n}=\hbar \omega \left(n+\frac{1}{2}\right), & \\ & \psi_{n}\left(x,t\right)=e^{i\frac{m}{\hbar }\dot{\alpha }x}\phi_{n}\left(x-\alpha \right), \end{array}$$

where $\phi_{n}\left(x\right)$ is the *n*th eigenvector of the stationary oscillator. The solution of the Schrödinger equation for our Hamiltonian is

(15) $$\psi \left(x,t\right)=\sum_{n}a_{n}e^{i\theta_{n}\left(t\right)}\psi_{n}\left(x,t\right),$$

with the Lewis–Riesenfeld phases

(16) $$\begin{array}{ccc} \theta_{n}\left(t\right) & = & -\frac{1}{\hbar }\int_{0}^{t}dt^{\prime }\left[\lambda_{n}+\frac{m}{2}\left({\dot{\alpha }}^{2}-\omega^{2}\alpha^{2}\right)\right]\\ & = & -\left(n+\frac{1}{2}\right)\omega t-\frac{m}{2\hbar }\int_{0}^{t}dt^{\prime }\left({\dot{\alpha }}^{2}-\omega^{2}\alpha^{2}\right)\\ & = & -\left(n+\frac{1}{2}\right)\omega t-G\left(t\right). \end{array}$$

The $f_{\alpha }\left(t\right)$ force with $c=x_{0}=0$ configuration guarantees that all dynamical modes $e^{i\theta_{n}\left(t\right)}\psi_{n}\left(x,t\right)$ end up at the original positions and at rest, $e^{i\theta_{n}\left(t_{f}\right)}\phi_{n}\left(x\right)$. At the same time,

(17) $$G\left(t_{f}\right)=-\frac{1}{2\hbar }\int_{0}^{t_{f}}dtf_{\alpha }\left(t\right)\alpha \left(t\right).$$

For the correspondent spin-down branch, we use $-f_{\alpha }$ and −α instead of $f_{\alpha }$ and α, respectively. The same common phase factor $G(t_{f})$ and Lewis–Riesenfeld phases $\theta (t_{f})$ at final time are obtained. Also, for any initial state $\psi_{↓}^{c=x_{0}=0}\left(x,0\right)=\psi_{↑}^{c=x_{0}=0}\left(x,0\right)$. We also have $\psi_{↓}^{c=x_{0}=0}\left(x,t_{f}\right)=\psi_{↑}^{c=x_{0}=0}\left(x,t_{f}\right)$, as we wanted.

What we do to actually design the $f_{\alpha }\left(t\right)$ driving force by inverse-engineering is first setting α(t) trajectories using sixth-order polynomials,

(18) $$\alpha \left(t\right)=\sum_{j=0}^{6}b_{j}\left(\frac{t}{t_{f}}\right).$$

We now apply the six boundary conditions. For the one free parameter left we impose an extra condition, the maximum displacement of the trap to be found at $t_{f}/2$: $\alpha (t_{f}/2)=M$. By selecting a larger $t_{f}$ and/or a larger value of *M*, we increase the area under the trajectory followed by the trapped ion, and so we increase the sensitivity of the interferometer.

From Equation (10) we derive a relation between the unknown force, *c*, and the the sensitivity *S*:

(19) $$c=\frac{\Delta \phi (t_{f})}{S}.$$

Once the trajectory is set, the corresponding driving force is obtained by solving the Newton equation. It is also noteworthy that the phase measurement is done through measurements of the populations. As populations are periodic oscillating functions of $\Delta \phi (t_{f})$ (see Equation (3)), we plot the populations as a function of *S*, so that *c* can be found from the oscillation period πℏ/c.

### 3.2. Alternative Scheme with No Rotation of the Trap

As we have seen above, a rotation of the trap is a formal but hardly practical solution which allows us to measure the unknown force *c*. However, in order to avoid any rotation, we may consider a different setting [10]. Here, instead of one fixed harmonic trap, we use two moving spin-dependent traps, complemented by spin- and time-dependent forces to compensate for inertial terms due to the motion of the traps. Another advantage of this new scheme is that, as the trapping potentials are not necessarily harmonic, we can trap ultracold neutral atoms rather than ions.

For each spin state, the Hamiltonians read

(20) $$\begin{array}{ccc} H^{↑↓} & = & \frac{p^{2}}{2m}-cx\mp \left[x-x_{0}\left(t\right)\right]f\left(t\right)+U\left[x\mp \alpha (t)\right]\\ & = & \frac{p^{2}}{2m}\mp f\left(t\right)x+\tilde{U}\left[x\mp \alpha (t)\right]+\Lambda^{↑↓}\left(t\right) \end{array}$$

where

$$\begin{array}{ccc} \Lambda^{↑↓}\left(t\right) & = & \pm f\left(t\right)x_{0}\left(t\right)\mp c\alpha \left(t\right)\\ \tilde{U}\left[x\mp \alpha (t)\right] & = & U\left[x\mp \alpha (t)\right]-\left[x\mp \alpha (t)\right]. \end{array}$$

Here, $U\left[x\mp \alpha (t)\right]$ are the trapping potentials moving alongside opposite trajectories ±α(t) which satisfy the boundary conditions $\alpha \left(t_{b}\right)=\dot{\alpha }\left(t_{b}\right)=\ddot{\alpha }\left(t_{b}\right)=0$ at $t_{b}=0,t_{f}$. We complement the trapping potentials with spin-dependent linear potential, $\mp \left[x-x_{0}\left(t\right)\right]f\left(t\right)$ that cross at $x_{0}\left(t\right)$.

The idea is inverse engineering f(t) to compensate inertial terms in the moving frame. We also have reorganized the Hamiltonians so that we separate purely time-dependent terms in $\Lambda^{↑↓}\left(t\right)$ and define effective trapping potentials $\tilde{U}$, which include the effect of the force *c*. To solve the dynamics we apply unitary transformations in the moving-frame interaction picture. The wavevectors in the interaction picture, $|\psi_{I}^{↑↓}\rangle$, and unitary operator, U, read

(21) $$|\psi_{I}^{↑↓}\rangle =U^{↑↓}|\psi^{↑↓}\rangle ,\;\;\;\;U=e^{\pm i\alpha p/\hbar }e^{\mp im\dot{\alpha }x/\hbar }.$$

The effective moving-frame Hamiltonians become

(22) $$\begin{array}{ccc} H_{I}^{↑↓} & = & U^{↑↓}H^{↑↓}{\left(U^{↑↓}\right)}^{†}+i\hbar {\dot{U}}^{↑↓}{\left(U^{↑↓}\right)}^{†}\\ & = & \frac{p^{2}}{2m}+\frac{1}{2}m{\dot{\alpha }}^{2}\mp \left(x\pm \alpha \right)f\left(t\right)+\tilde{U}\left(x\right)\\ & & \pm f\left(t\right)x_{0}\left(t\right)\mp c\alpha \pm \left(x\pm \alpha \right)m\ddot{\alpha }. \end{array}$$

Again, the compensating $f_{\alpha }\left(t\right)$ is designed inversely from the Newton equation for α(t) trajectories, $\ddot{\alpha }\left(t\right)=f_{\alpha }\left(t\right)/m$. Taking this into account we could restructure the Hamiltonians

(23) $$\begin{array}{ccc} & H_{I}^{↑↓}=H_{I,0}+F^{↑↓}\left(t\right),\;\;\;\;H_{I,0}=\frac{p^{2}}{2m}+\tilde{U}\left(x\right) & \\ & F^{↑↓}\left(t\right)=\frac{1}{2}m{\dot{\alpha }}^{2}\mp f\left(t\right)x_{0}\left(t\right)\mp c\alpha \left(t\right). \end{array}$$

This construction largely facilitates the formal solutions of the dynamics (see Ref. [10] for detailed calculations). The wavefunctions in the laboratory frame are

(24) $$\psi^{↑↓}\left(x,t\right)=e^{im\dot{\alpha }x/\hbar }\exp\left(-i\int_{0}^{t}F^{↑↓}\left(t^{\prime }\right)dt^{\prime }/\hbar \right)\Phi \left(x\mp \alpha ,t\right),$$

where $|\psi_{I,0}^{↑↓}\left(t\right)\rangle =|\Phi \left(t\right)\rangle =e^{-iH_{I,0}t/\hbar }$ and $\langle x|e^{\mp i\alpha p/\hbar }|\Phi \rangle =\Phi \left(x\mp \alpha \right)$. For constant $x_{0}$ the overlap at final time takes the form

(25) $$\langle \psi^{↓}\left(t_{f}\right)|\psi^{↑}\left(t_{f}\right)\rangle =\exp\left(2ic\int_{0}^{t_{f}}\alpha \left(t\right)dt/\hbar \right)$$

so that the phase differential at the final time is proportional to *c* with a controllable sensitivity

(26) $$S=\frac{2}{\hbar }\int_{0}^{t_{f}}\alpha \left(t\right)dt.$$

It is significant that the phase differential within this scheme is independent of $x_{0}$. Thus, no rotation of the trap is required. This, together with the fact that ultracold neutral atoms can be used, makes this proposal more practical and more broadly applicable.

These results might also be connected with the inverse engineering of the trap trajectories based on Lewis–Riesenfeld invariants of motion. For the branch Hamiltonians $H^{↑↓}$ we can find invariants of motion of the form

(27) $$I^{↑↓}=\frac{1}{2m}{\left(p\mp m\dot{\alpha }\right)}^{2}+\tilde{U}\left(x\mp \alpha \right),$$

whose eigenvalues, $\lambda_{n}$, are the eigenvalues of $H_{I,0}$. By solving

(28) $$\frac{d\theta_{n}^{↑↓}\left(t\right)}{dt}=\frac{1}{\hbar }\langle \psi_{n}^{↑↓}\left(t\right)|i\hbar \frac{\partial }{\partial t}-H^{↑↓}|\psi_{n}^{↑↓}\left(t\right)\rangle$$

we get the Lewis–Riesenfeld phases

(29) $$\theta_{n}^{↑↓}\left(t\right)=\frac{-1}{\hbar }\int_{0}^{t}\left[\lambda_{n}+F^{↑↓}\left(t^{\prime }\right)\right]dt^{\prime },$$

where we set $\theta_{n}^{↑↓}\left(0\right)=0$. The branch wavefunctions read $\psi^{↑↓}\left(x,t\right)=\sum_{n}c_{n}e^{i\theta_{n}^{↑↓}\left(t\right)}\psi_{n}\left(x,t\right)$. Summing over all *n* states we recover the expression in Equation (24).

## 4. Measuring the Mass of an Ion

In this section we present a new protocol whose goal is not to measure weak unknown forces but rather is aimed at providing an alternative way of measuring the mass of an ion trapped in a fixed harmonic potential.

We consider, again, the Hamiltonians of Equation (4) as our setting still consists on an ion trapped in a harmonic potential and subjected to a spin-dependent force $f(t,\sigma_{z})$. The phase differential is, therefore, still given by Equation (8). However, the current proposal differs from the previous one. There, the spin-dependent forces were applied at a constant $x_{0}$ point, whose “natural” choice was the minimum of the trapping potential.

Alternatively, in this new proposal, we consider that these spin-dependent driving forces are generated by a “shaken” optical lattice [16,17]. The potential of this optical lattice may be considered of the form $V_{0}sin^{2}\left(kx+\gamma \left(t\right)\right)$. A linear Taylor series of the optical potential around x=0 is a good approximation and would generate homogeneous driving forces [8]. By “shaken” optical lattice, we mean phase modulated. Changing the value of γ(t) we might move the lattice from left to right and the other way around in a periodic movement, which induces an oscillating $x_{0}\left(t\right)=\beta +A_{0}sin\left(\omega_{0}t+\gamma_{0}\right)$ around the pivotal point β.

Introducing $x_{0}\left(t\right)$ in Equation (8), we get ($\gamma_{0}=0$ is considered for simplicity):

(30) $$\begin{array}{ccc} \Delta \phi (t_{f}) & = & \frac{2}{\hbar }\left[c-\beta m\omega^{2}\right]\int_{0}^{t_{f}}\alpha \left(t\right)dt\\ & & -\frac{2A_{0}m}{\hbar }\left[\omega^{2}-\omega_{0}^{2}\right]\int_{0}^{t_{f}}\alpha \left(t\right)\sin\left(\omega_{0}t\right)dt. \end{array}$$

We let *c* act on the ion even at t≤0, which means that $\beta =c/m\omega^{2}$. The phase differential at final time reads

(31) $$\Delta \phi \left(t_{f}\right)=\frac{2A_{0}m}{\hbar }\left[\omega_{0}^{2}-\omega^{2}\right]I\left(\omega_{0}\right)=S^{*}\cdot m,$$

where $I\left(\omega_{0}\right)=\int_{0}^{t_{f}}\alpha \left(t\right)\sin\left(\omega_{0}t\right)dt$.

As we can see, $\Delta \phi (t_{f})$ is proportional to the mass of the ion, where $S^{*}=\frac{2A_{0}}{\hbar }\left[\omega_{0}^{2}-\omega^{2}\right]I\left(\omega_{0}\right)$ would be the sensitivity within this new setting. If we had accurate control of both the amplitude, $A_{0}$, and the frequency, $\omega_{0}$, of the oscillation, we could measure *m* unambiguously from the periodicity 2π/m of the populations $P_{↑↓}\left(t_{f}\right)$ given in Equation (3) as a function of $S^{*}$. Please note that $S^{*}$ depends on $I(w_{0})$, which in turn depends on the α(t) trajectories, and on the process time. Thus, the sensitivity may be adjusted in principle at will, subjected to technical limitations.

We would like to remark that this is just a theoretical possibility and that there exist broadly extended very accurate methods to measure the mass of the ions, for example, measurements of cyclotron frequencies of single ions in Penning traps with a precision at $10^{-10}$ level or better [20] or using cryogenic multi-Penning traps with a relative precision of a few parts per trillion [21].

### 4.1. Trajectories and Forces

We will first show how to set the trajectory α(t). Once α(t) is designed, we inverse engineer the corresponding driving force by solving the Newton Equation (7). Remember α(t) are solutions of the Newton equation satisfying the boundary conditions $\alpha \left(t_{b}\right)=\dot{\alpha }\left(t_{b}\right)=\ddot{\alpha }\left(t_{b}\right)=0$ for $t_{b}=0,t_{f}$. From Equation (31) we can derive a relation between the mass of the ion and the expression $I(\omega_{0})$ which, as commented above, depends on α(t):

(32) $$m=\frac{\hbar \Delta \phi (t_{f})}{2A_{0}\left[\omega_{0}^{2}-\omega^{2}\right]I\left(\omega_{0}\right)}.$$

The larger the value of $I(\omega_{0})$, the smaller the value of *m* that we could measure, and the higher accuracy we could get. Although different strategies might be followed, we consider trigonometric polynomials of order *k* to design the trajectories,

(33) $$\alpha_{k}\left(t\right)=\sum_{n=1}^{k}b_{n}\sin\left[\frac{(2n-1)\pi t}{t_{f}}\right],$$

so that just by construction, and independently of the $b_{n}$ coefficients, four of the required six boundary conditions are fulfilled: $\alpha_{k}\left(t_{b}\right)=\ddot{\alpha_{k}}\left(t_{b}\right)=0$.

In the following we will present several types of trajectories varying the grade of the trigonometric polynomials. For each of these trajectories we inverse engineer the driving force and analyze the value of $I(\omega_{0})$.

#### 4.1.1. Trajectories with Two Terms

Let us now consider k=2 in Equation (33).

(34) $$\alpha_{2}\left(t\right)=b_{1}\sin\left(\frac{\pi t}{t_{f}}\right)+b_{2}\sin\left(\frac{3\pi t}{t_{f}}\right)=\frac{3\alpha_{max}}{4}\sin\left(\frac{\pi t}{t_{f}}\right)-\frac{\alpha_{max}}{4}\sin\left(\frac{3\pi t}{t_{f}}\right).$$

Here, we have first applied the remaining boundary conditions, $\dot{\alpha_{2}}\left(t_{b}\right)=0$, so that $b_{1}=-3b_{2}$. Also, in order to adjust the free parameter $b_{2}$, we impose an extra condition [8] $\alpha_{2}\left(t_{f}/2\right)=\alpha_{max}$, from whom $b_{2}=-\alpha_{max}/4$ is obtained.

The value $\alpha_{max}$ will be the maximum displacement of the ion with respect to its original position. For such a trajectory, we can inverse engineer its corresponding driving force.

**Figure 1:**
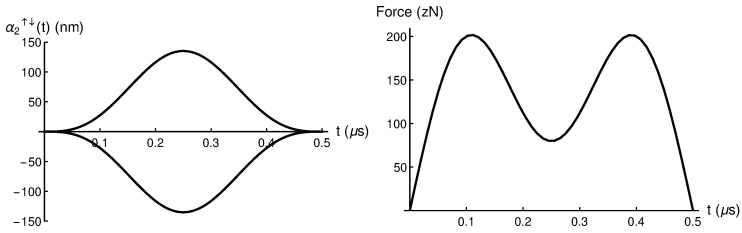
(**Left**) $\alpha_{2}^{↑↓}\left(t\right)$ spin-up and spin-down trajectories, and (**Right**) the corresponding driving force $f_{\alpha_{2}}\left(t\right)$ obtained via Newton equation for a selected $\alpha_{max}=135$ nm. Please note that zN refers to zeptoNewtons (1 zN = $10^{-21}\;N$).

In Figure 1 we plot $\alpha_{2}^{↑↓}\left(t\right)$ and its corresponding driving force for a selected $\alpha_{max}=135$ nm. This maximum displacement has been selected so that the required driving forces (≈200 zN) are experimentally achievable. See [8] for further details.

We can, also, introduce the trajectory $\alpha_{2}\left(t\right)$ in $I\left(\omega_{0}\right)=\int_{0}^{t_{f}}\alpha \left(t\right)\sin\left(\omega_{0}t\right)dt$:

(35) $$I\left(\omega_{0}\right)=\frac{3\alpha_{max}\left(t\right)}{4}I_{1}\left(\omega_{0}\right)-\frac{\alpha_{max}\left(t\right)}{4}I_{3}\left(\omega_{0}\right),$$

where

(36) $$I_{i}\left(\omega_{0}\right)=\frac{i\cdot \pi \cdot t_{f}\sin\left(\omega_{0}t_{f}\right)}{\left(i\cdot \pi -\omega_{0}t_{f}\right)\left(i\cdot \pi +\omega_{0}t_{f}\right)}.$$

**Figure 2:**
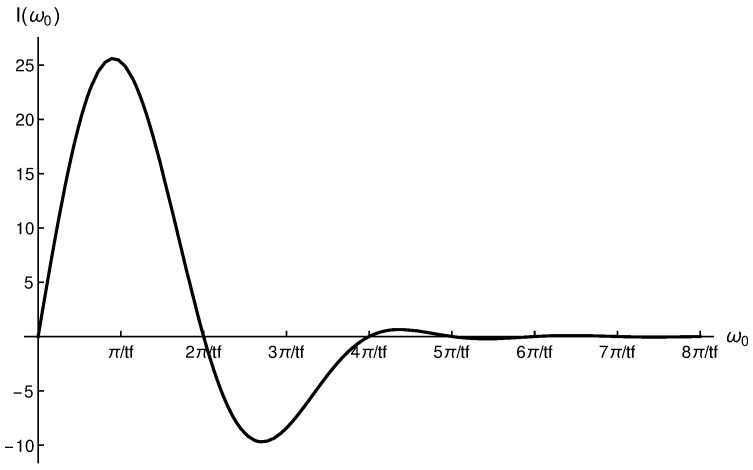
$I(\omega_{0})$ for a $\alpha_{2}\left(t\right)$ trajectory which includes $\sin(\pi /t_{f})$ and $\sin(3\pi /t_{f})$ terms. Maximum peak is obtained at $\omega_{0}=\pi /t_{f}$, whereas the minimum is located at $\omega_{0}=2.70482\times \frac{\pi }{t_{f}}$. A value of $\alpha_{max}=135$ nm has been considered.

In Figure 2 we plot the $I(\omega_{0})$ function for a $\alpha_{2}\left(t\right)$ trajectory with $\alpha_{max}=135$ nm.

For values of $\omega_{0}=2k\frac{\pi }{t_{f}},k\in Z$, we can see that $I_{1}\left(\omega_{0}\right)=I_{2}\left(\omega_{0}\right)=0$. Therefore, $\Delta \phi (t_{f})=0$. On the other hand, the maximum value of $I(\omega_{0})$ is found for $\omega_{0}=\frac{\pi }{t_{f}}$, where $I\left(\omega_{0}=\frac{\pi }{t_{f}}\right)=\frac{3}{8}\alpha_{max}t_{f}$. Thus,

(37) $$\Delta \phi \left(t_{f}\right)=\frac{2A_{0}m}{\hbar }\left[\frac{\pi^{2}}{t_{f}^{2}}-\omega_{0}^{2}\right]\times \frac{3}{8}\alpha_{max}t_{f}.$$

The larger $\alpha_{max}$ and $t_{f}$, the larger phase differential we get, and the smaller masses we could measure. We can also control the amplitude of the oscillation $A_{0}$ of the “shaken” optical lattice and the frequency ω of the trapping harmonic potential.

Other kind of trajectories containing just two terms might be considered. For example,

(38) $$\alpha_{2b}\left(t\right)=b_{1}\sin\left(\frac{\pi t}{t_{f}}\right)+b_{3}\sin\left(\frac{5\pi t}{t_{f}}\right)=\frac{5\alpha_{max}}{4}\sin\left(\frac{\pi t}{t_{f}}\right)-\frac{\alpha_{max}}{4}.$$

**Figure 3:**
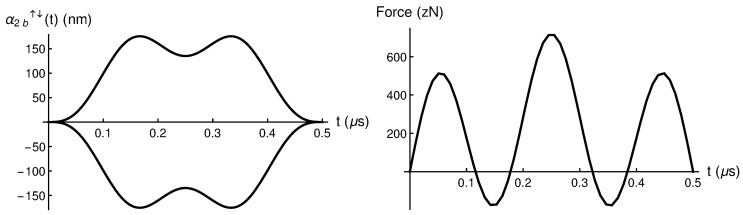
(**Left**) $\alpha_{2b}^{↑↓}\left(t\right)$ spin-up and spin-down trajectories, and (**Right**) the corresponding driving force $f_{\alpha_{2b}}\left(t\right)$ obtained via Newton equation, for a selected $\alpha_{max}=135$ nm. Please note that zN refers to zeptoNewtons (1 zN = $10^{-21}\;N$).

Here we follow the same procedure than in the previous case and impose $\dot{\alpha_{2b}}\left(t_{b}\right)=0$ and $\alpha_{2b}\left(t_{f}/2\right)=\alpha_{max}$ so that we get $b_{1}=-5b_{3}=\frac{5}{4}\alpha_{max}$. In Figure 3 we plot $\alpha_{2b}^{↑↓}\left(t\right)$ and its corresponding $f_{\alpha_{2b}}\left(t\right)$ force for a selected $\alpha_{max}=135$ nm. Note that we need a highest force of ≈700 zN compared to the previous case where the highest required force was ≈200 zN.

**Figure 4:**
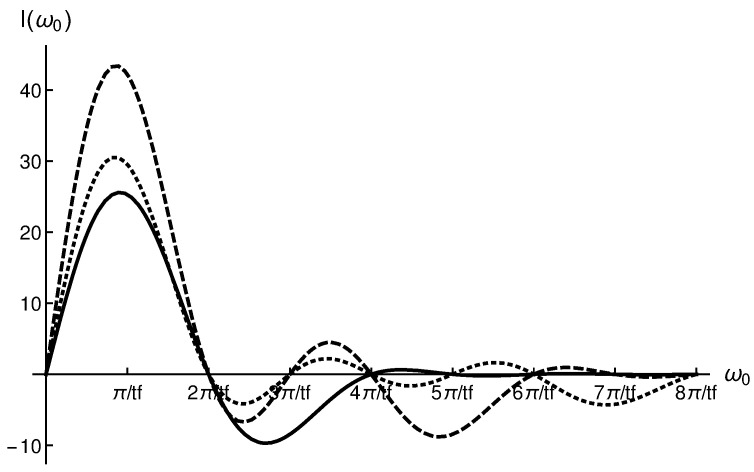
$I(\omega_{0})$ for $\alpha_{2}\left(t\right)$, $\alpha_{2b}\left(t\right)$ (dashed), and $\alpha_{2c}\left(t\right)$ (dotted), respectively. A value of $\alpha_{max}=135$ nm has been considered. Please note that the maximum value of $I(\omega_{0})$ for all the trajectories is obtained at $\omega_{0}=\Pi /t_{f}$. With the $\alpha_{2b}\left(t\right)$ trajectories we get the highest value.

The value of $I(\omega_{0})$ for $\alpha_{2b}\left(t\right)$ is

(39) $$I\left(\omega_{0}\right)=\frac{5\alpha_{max}}{4}I_{1}\left(\omega_{0}\right)+\frac{\alpha_{max}}{4}I_{5}\left(\omega_{0}\right).$$

where $I_{i}\left(\omega_{0}\right)$ is given by Equation (36). The maximum of $I(\omega_{0})$ is $I\left(\omega_{0}=\frac{\pi }{t_{f}}\right)=\frac{5}{8}\alpha_{max}t_{f}$. We can, very similarly, include any desired two terms in our trajectory. In Figure 4 we plot the $I(\omega_{0})$ for $\alpha_{2}\left(t\right)$, $\alpha_{2b}\left(t\right)$ and $\alpha_{2c}\left(t\right)$ trajectories considering $\alpha_{max}=135$ nm. The $\alpha_{2c}\left(t\right)=b_{1}\sin\left(\frac{\pi t}{t_{f}}\right)+b_{4}\sin\left(\frac{7\pi t}{t_{f}}\right)$ trajectory is also designed imposing $\dot{\alpha_{2c}}\left(t_{b}\right)=0$ and $\alpha_{2c}\left(t_{f}/2\right)=\alpha_{max}$.

The maximum value of $I(\omega_{0})$, which is located at $\omega_{0}=\pi /t_{f}$ for all the trajectories, is highest for the $\alpha_{2b}\left(t\right)$ trajectory. Similarly, we have also considered $\alpha_{2d}\left(t\right)$ trajectories (which would include $\sin(\pi /t_{f})$ and $\sin(9\pi /t_{f})$ terms), $\alpha_{2e}\left(t\right)$ trajectories (which would include $\sin(\pi /t_{f})$ and $\sin(11\pi /t_{f})$ terms), and so on, and this maximum is never overcome.

#### 4.1.2. Trajectories with More Terms

Let us now consider k=3 in Equation (33)

(40) $$\alpha_{3}\left(t\right)=c_{1}\sin\left(\frac{\pi t}{t_{f}}\right)+c_{2}\sin\left(\frac{3\pi t}{t_{f}}\right)+c_{3}\sin\left(\frac{5\pi t}{t_{f}}\right).$$

**Figure 5:**
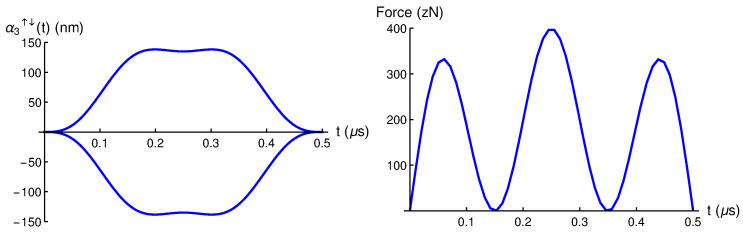
(**Left**) $\alpha_{3}^{↑↓}\left(t\right)$ spin-up and spin-down trajectories, and (**Right**) the corresponding driving force f(t) obtained via Newton equation, for a selected A=135 nm.

We first impose the boundary conditions $\dot{\alpha_{3}}\left(t_{b}\right)=0$, obtaining that $c_{1}=-3c_{2}-5c_{3}$. As we have now two parameters left, we need to impose two extra conditions. For example we could require our trajectory to fulfill $\alpha_{3}\left(t_{f}/2\right)=A$ and $\alpha_{3}\left(t_{f}/4\right)=\frac{\sqrt{2}}{2}A$. Under these conditions $c_{2}=c_{3}=-A/8$, so $c_{1}=A$. In Figure 5 we plot $\alpha_{3}^{↑↓}\left(t\right)$ and its corresponding driven force for A=135 nm.

For this trajectory we have that

(41) $$I\left(\omega_{0}\right)=AI_{1}\left(\omega_{0}\right)-\frac{A}{8}\left(I_{3}\left(\omega_{0}\right)+I_{5}\left(\omega_{0}\right)\right)$$

where $I_{i}\left(\omega_{0}\right)$ is given by Equation (36). Again, its maximum peak is obtained at $\omega_{0}=\pi /t_{f}$ where $I\left(\omega_{0}=\pi /t_{f}\right)=\frac{1}{2}\alpha_{max}t_{f}$.

**Figure 6:**
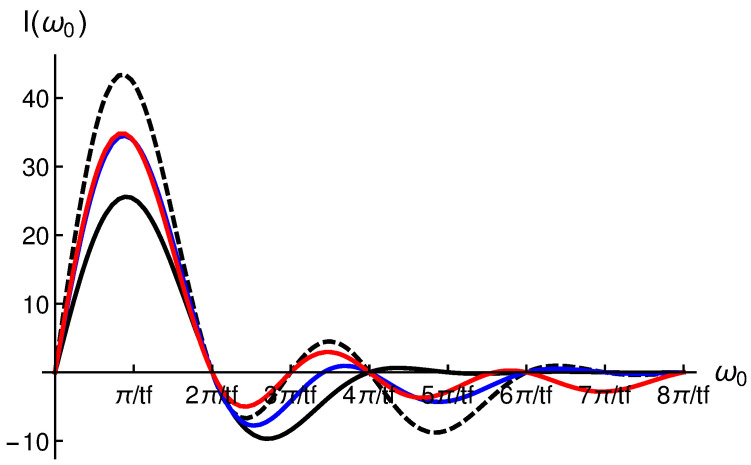
(Line/color). $I(\omega_{0})$ for $\alpha_{2}\left(t\right)$ (black), $\alpha_{2b}\left(t\right)$ (black, dashed), $\alpha_{3}\left(t\right)$ (blue) and $\alpha_{4}\left(t\right)$ (red), respectively. A value of $\alpha_{max}=135$ nm has been considered.

In Figure 6 we plot $I(\omega_{0})$ for several trajectories: $\alpha_{2}\left(t\right)$, $\alpha_{2b}\left(t\right)$, $\alpha_{3}\left(t\right)$ and $\alpha_{4}\left(t\right)$. In order to design

$$\alpha_{4}\left(t\right)=\sum_{n=1}^{4}b_{n}\sin\left[\frac{(2n-1)\pi t}{t_{f}}\right]$$

we impose the following conditions: $\dot{\alpha_{4}}\left(t_{b}\right)=0$, $\alpha_{4}\left(t_{f}/2\right)=\alpha_{max}$, $\alpha_{4}\left(t_{f}/3\right)=\frac{\sqrt{3}}{2}\alpha_{max}$ and $\alpha_{4}\left(t_{f}/6\right)=\frac{1}{2}\alpha_{max}$.

We can see that maximum value of $I(\omega_{0}=\frac{\pi }{t_{f}})$ is obtained for $\alpha_{2b}\left(t\right)$ trajectory, although higher driving forces than for $\alpha_{2}\left(t\right)$ and $\alpha_{3}\left(t\right)$ are required. We have also considered $\alpha_{5}\left(t\right)$, $\alpha_{6}\left(t\right)$ and so on, and the values of $I(\omega_{0}=\frac{\pi }{t_{f}})$ are very close to the one obtained for $\alpha_{3}\left(t\right)$, while the corresponding driving forces are significantly larger. Thus, designing trajectories with just two terms seems to be the smartest choice.

## 5. Discussion

In previous work [8], we presented the theory to perform STA-mediated guided interferometry on a trapped ion with controllable driving spin-dependent homogeneous forces. Specifically, we considered the measurement of an unknown weak force using an ion trapped in a harmonic potential. However, a rotation of the trap from a perpendicular position was required. In order to avoid this rotation we developed a second scheme [10]. Here we used two moving spin-dependent traps complemented by homogeneous spin- and time-dependent compensating forces, which is one of the ways to implement STA-driven fast transport.

In this paper, besides a brief review of these two works, we revisit the first scheme and propose a different protocol aimed at measuring the mass of the trapped ion. Operationally, this new proposal, while keeping all the advantages of the previous one, differs from it in considering that the driving force is generated via a “shaken” optical lattice, which leads to an oscillating $x_{0}\left(t\right)$.

The sensitivity of the interferometer depends, among other factors, on the α(t) trajectory followed by the driven ion. We have shown how to design trajectories and inverse-engineer the driving forces required to get those trajectories. By doing so we get control of the sensitivity, provided we also have control of the amplitude of the oscillation.

Several extensions of this work are possible. For example, we can consider that the driving force f(t) may suffer from some noisy deviation from the ideal value. That noisy deviation may be treated as in [22]. We could also numerically calculate the trajectory which maximizes the phase differential at final time. This leads to a constrained optimization problem where we aim to maximize the objective functional $I\left(\omega_{0}\right)=\int_{0}^{t_{f}}\alpha \left(t\right)\sin\left(\omega_{0}\left(t\right)\right)dt$, under the integral constraint $\int_{0}^{t_{f}}\alpha \left(t\right)dt=\mathrm{constant}$ in order to keep constant the area under the trajectory of the ion. Finally, these results could be extended to oscillating forces.

## Data Availability

The original contributions presented in this study are included in the article.
